# Procalcitonin for the diagnosis of postoperative bacterial infection after adult cardiac surgery: a systematic review and meta-analysis

**DOI:** 10.1186/s13054-024-04824-3

**Published:** 2024-02-07

**Authors:** Davide Nicolotti, Silvia Grossi, Valeria Palermo, Federico Pontone, Giuseppe Maglietta, Francesca Diodati, Matteo Puntoni, Sandra Rossi, Caterina Caminiti

**Affiliations:** 1 Department of Anesthesia and Intensive Care Medicine, University Hospital of Parma, Parma, Italy; 2 Clinical and Epidemiological Research Unit, University Hospital of Parma, Parma, Italy

## Abstract

**Background and aims:**

Patients undergoing cardiac surgery are subject to infectious complications that adversely affect outcomes. Rapid identification is essential for adequate treatment. Procalcitonin (PCT) is a noninvasive blood test that could serve this purpose, however its validity in the cardiac surgery population is still debated. We therefore performed a systematic review and meta-analysis to estimate the accuracy of PCT for the diagnosis of postoperative bacterial infection after cardiac surgery.

**Methods:**

We included studies on adult cardiac surgery patients, providing estimates of test accuracy. Search was performed on PubMed, EmBase and WebOfScience on April 12th, 2023 and rerun on September 15th, 2023, limited to the last 10 years. Study quality was assessed with the QUADAS-2 tool. The pooled measures of performance and diagnostic accuracy, and corresponding 95% Confidence Intervals (CI), were calculated using a bivariate regression model. Due to the variation in reported thresholds, we used a multiple-thresholds within a study random effects model for meta-analysis (diagmeta R-package).

**Results:**

Eleven studies were included in the systematic review, and 10 (2984 patients) in the meta-analysis. All studies were single-center with observational design, five of which with retrospective data collection. Quality assessment highlighted various issues, mainly concerning lack of prespecified thresholds for the index test in all studies. Results of bivariate model analysis using multiple thresholds within a study identified the optimal threshold at 3 ng/mL, with a mean sensitivity of 0.67 (0.47–0.82), mean specificity of 0.73 (95% CI 0.65–0.79), and AUC of 0.75 (IC95% 0.29–0.95). Given its importance for practice, we also evaluated PCT’s predictive capability. We found that positive predictive value is at most close to 50%, also with a high prevalence (30%), and the negative predictive value was always > 90% when prevalence was < 20%.

**Conclusions:**

These results suggest that PCT may be used to help rule out infection after cardiac surgery. The optimal threshold of 3 ng/mL identified in this work should be confirmed with large, well-designed randomized trials that evaluate the test’s impact on health outcomes and on the use of antibiotic therapy.

*PROSPERO Registration number* CRD42023415773. Registered 22 April 2023.

**Graphical abstract:**

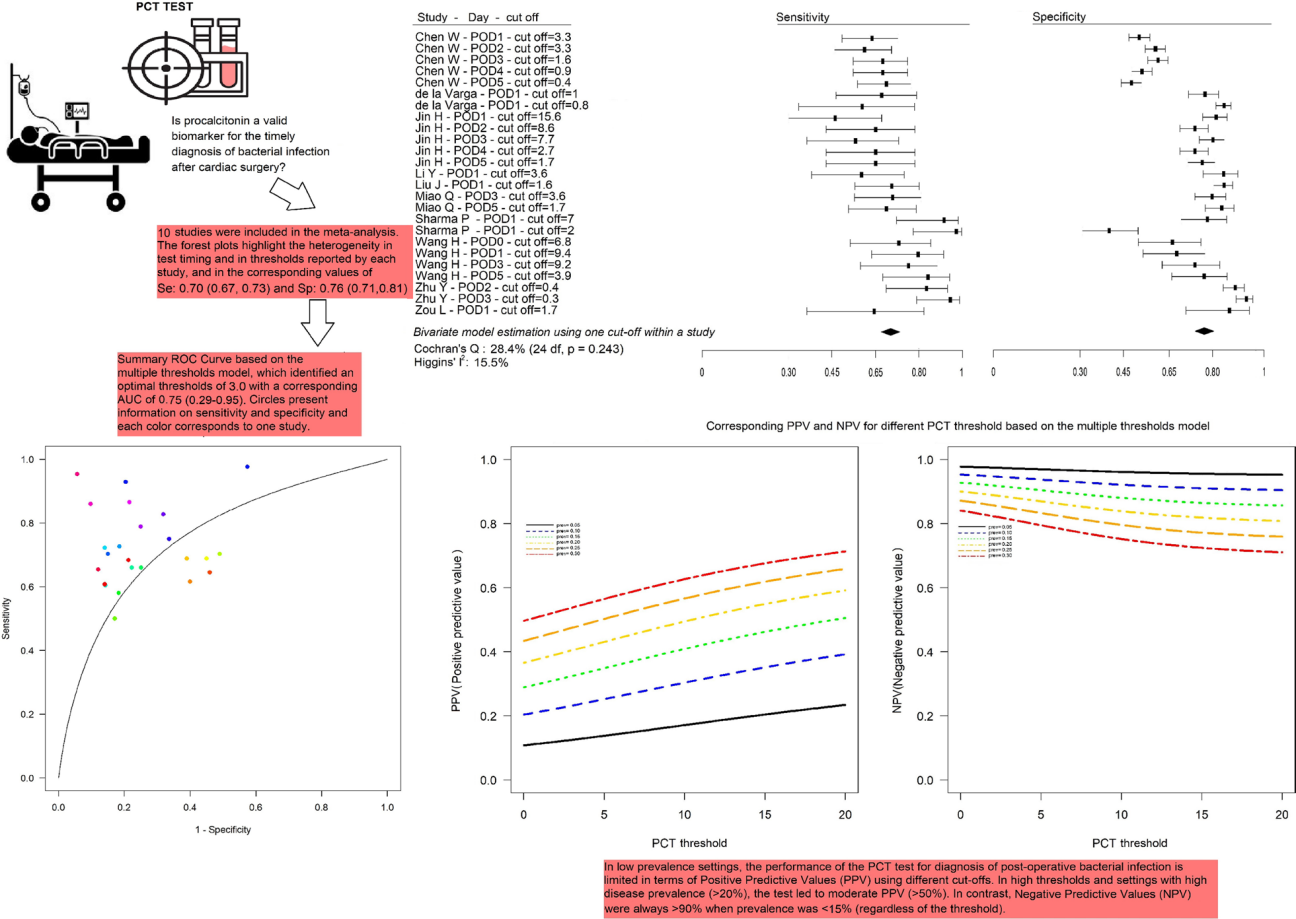

**Supplementary Information:**

The online version contains supplementary material available at 10.1186/s13054-024-04824-3.

## Introduction

One of the major complications that can occur after cardiac surgery is postoperative infection, including pneumonia, surgical site infection, Clostridioides difficile colitis, and blood stream infections [1]. These complications have a reported incidence of 5–21%, and are associated with unfavorable outcomes, such as delayed hospital discharge, prolonged recovery, and a five-time increase in the postoperative death rate [2]. Timely and accurate diagnosis of postsurgical infective complications is essential, both to ensure prompt treatment to affected patients, and to avoid the use of antibiotics when not necessary [3–5]. Unfortunately this task can be challenging, since many typical signs of infection are nonspecific and common in the critically ill [4, 5]. Specifically, cardiac surgery with cardiopulmonary bypass (CPB) induces an acute inflammatory response that may lead to a systemic inflammatory response syndrome (SIRS), which may mimic the typical clinical and biological manifestations of infection [6].

Conventional diagnostic tests for infection (such as blood cultures and inflammatory markers) have important limitations, particularly concerning suboptimal sensitivity and specificity [7, 8]. In particular, microbiological cultures, generally considered the most reliable diagnostic method for identification of pathogens, provide important information on type of microorganism and susceptibility toward antibiotic treatment, but test results take a long time to be available, and are characterized by a high proportion of false negatives [9].

In the quest for a highly specific test yielding rapid results, host biological biomarkers are receiving increasing attention [9]. One of these is procalcitonin (PCT), the peptide precursor to calcitonin. PCT is released from thyroid C glands at very low levels under normal physiological conditions, but its synthesis can be greatly increased in response to infection and inflammation [8]. The use of PCT as a diagnostic marker for infection has been established in specific settings; the United States Food and Drug Administration has approved its use for initiating or discontinuing antibiotics in lower respiratory tract infections and for discontinuing antibiotics in patients with sepsis [8]. However, the use of PCT for prescribing antimicrobial medications in septic patients has been questioned and is not recommended by recent guidelines [10, 11]. Concerning applications in surgery, some meta-analyses have investigated the diagnostic accuracy of PCT for postoperative infection on different populations, such as major gastrointestinal surgery [12], liver transplantation [13], colorectal surgery [14], and solid organ transplantation [15], reporting mixed results. To our knowledge, the only existing meta-analysis on the diagnostic accuracy of PCT for infection post-cardiac surgery including adult patients was performed in 2021 by Li et al. [16]. This work included 14 studies published between 2000 and 2017, and considered both children (six articles) and adults (eight articles). The authors concluded that PCT was a promising marker for the diagnosis of sepsis for cardiac surgery patients. However, the inclusion of children may have amplified the effect, since in pediatric patients mean postoperative PCT values are markedly higher after cardiac surgery [17].

Based on the above considerations, we performed a systematic review and meta-analysis to evaluate the accuracy of PCT for the diagnosis of postoperative bacterial infection in patients undergoing cardiac surgery. We restricted inclusion to studies on adult subjects and applied stringent eligibility criteria for the diagnosis of the target condition, to reduce heterogeneity.

## Methods

Before commencing this work, the PROSPERO database [18] was searched in March 2023, to identify any ongoing review with the same study question, but none was found. This review was designed and conducted following the Preferred Reporting for Systematic reviews and Meta-Analyses (PRISMA) [19] and the Preferred reporting items for systematic review and meta-analysis of diagnostic test accuracy studies (PRISMA-DTA) [20] guidelines. The protocol was registered with PROSPERO (CRD42023415773) on 22 April 2023.

### Criteria for considering studies for this review

#### Types of studies

We considered studies evaluating the diagnostic accuracy of PCT (index test) for postoperative bacterial infection (target condition) among adult patients undergoing cardiac surgery. Studies were eligible if they produced estimates of test accuracy or provided 2 × 2 data (true positive (TP), false positive (FP), true negative (TN), false negative (FN)) from which estimates for the primary objective could be computed.

We excluded studies with fewer than 10 participants and single case reports, as well as literature reviews, editorial material, and meeting abstracts. Inclusion was restricted to reports published from January 1st, 2013 to September 15th, 2023, to better reflect the current situation, where improvements in standards of care have led to a decrease in surgery-related stress, and thus of the occurrence of SIRS, which may be misclassified as bacterial infection.

#### Population eligibility

Studies had to concern adult patients (age ≥ 18 years) undergoing surgery of the heart or ascending aorta/aortic arch, with or without the use of CPB, regardless of type of surgical access site, and without infection before surgery. Subjects undergoing transcatheter interventions were also excluded.

#### Index test

PCT, measured at least once after surgery using any kit and method of assay. We reported these index tests as positive or negative on the basis of study threshold cutoffs.

#### Target condition

Any postoperative bacterial infection. Diagnosis had to be made according to clearly defined criteria, such as the ones established by the Centers for Disease Control [21], to ensure that a predetermined reference standard was used.

#### Search strategy and literature selection

The search strategies were developed by an information specialist (FD), in close collaboration with the clinicians in the research team. MedLine (PubMed platform), EmBase, and Web Of Science Clarivate were searched, with no language restrictions, from 2013 to present. The original search was performed on April 12th, 2023, and rerun on September 15th, 2023. A “backwards” snowball search was conducted on the references of systematic reviews and relevant papers. The full search strategies for each database together with notes on their development are provided in Additional file 1: Table S1.

Title and abstract screening was performed independently by two reviewers (DN and VP) using the Rayyan platform [22] and discrepancies were resolved by consulting a third reviewer (CC). Next, two reviewers (SG and FP) independently examined the full texts of the screened publications to determine eligibility with respect to protocol criteria. Again, disagreements were resolved by a third independent reviewer (CC).

#### Data extraction

Information on diagnostic accuracy from eligible papers was extracted by two researchers independently (CC and GM), using a Microsoft Excel form, and disagreements were resolved through discussion, involving a third reviewer when necessary (MP).

When the numbers of TP, FP, TN, and FN were not available, we extracted them based on the provided indices of Sensitivity (Se), Specificity (Sp), and sample size values.

Study investigators were contacted when data confirmation was needed.

#### Assessment of methodological quality

Methodological quality of included studies was assessed using the Quality Assessment of Diagnostic Accuracy Studies (QUADAS-2) checklist [23], recommended by the Cochrane collaboration for the quality assessment of diagnostic studies. The QUADAS-2 tool comprises four domains: patient selection, index test, reference standard, flow and timing, and enables to rate both risk of bias of included studies and their applicability to the review question. Signaling questions are provided to help reach judgments on risk of bias. Quality assessment was performed independently by two reviewers (CC and FD), and conflicts resolved by a third reviewer (MP). Risk of bias in QUADAS-2 is judged as “low”, “high”, or “unclear”. Following the instrument’s manual [24], risk of bias was judged “low” when all signaling questions for a domain were answered “yes”. If any signaling question was answered “no”, reviewers discussed the potential for bias. We did not construct funnel plots, because in meta-analyses of diagnostic studies, statistical tests based on funnel plot asymmetry do not allow to discriminate between publication bias and other sources of asymmetry, like the effect of including multiple thresholds [25].

#### Statistical analysis and data synthesis

We planned to perform the meta-analysis if four or more studies were available. Classification tables (TP, FP, TN, FN) were extracted or reconstructed to calculate the performance of the index biomarker. The included studies contributed varying numbers of test days and postoperative thresholds, as well as different thresholds on the same day. For the analyses, we extracted accuracy data on all cut-off points for which the data was available or could be calculated.

Estimates of SE, SP, and corresponding 95% Confidence Intervals (CI) for each study were graphically illustrated in forest plots.

The pooled diagnostic accuracy (Se, Sp, positive and negative likelihood ratios (PLR and NLR), diagnostic odds ratio (DOR)), were calculated using a bivariate model [26] accounting for within- and between-study variance. This model creates a link between the range of thresholds and the respective pairs of sensitivity and specificity, and thus allows to identify thresholds at which the test is likely to perform best. We used PLR and NLR as an indication of clinical informativeness. A PLR greater than 1 indicates that a positive test is associated with an increase in the likelihood of an infection being present. A NLR less than 1 indicates that a negative test is associated with a decrease in the likelihood of an infection. Furthermore, likelihood ratios above 10 and below 0.1 are considered to provide strong evidence to rule in or rule out diagnoses, respectively[27]. The DOR is a measure of discriminatory test performance that compares the odds of positivity in a disease state to the odds of positivity in a non-disease state, with higher values indicating better performance [28]. Bivariate model analysis using multiple thresholds within a study enabled to determine an optimal threshold and a Summary Receiver Operating Characteristic (SROC) curve and the corresponding Area Under the Curve (AUC) [29]. Since heterogeneity is to be expected in meta-analyses of diagnostic test accuracy, random effects methods were used. Furthermore, by considering the varying thresholds per day, interaction terms (threshold* day) were added and analyzed with the bivariate model analysis using multiple thresholds within a study.

Finally, for clinical practice, it is necessary to know the probability of a patient having a postoperative bacterial infection or not when the PCT test result exceeds a certain threshold. To address this issue, we also used the bivariate multiple-threshold model and calculated Negative Predictive Value (NPV) and Positive Predictive Value (PPV), relative to a simulated range of threshold values (1 to 5) for different prevalence levels (5–30%).

All Statistical analysis were performed with R for Windows (Version 4.2.2; R Foundation for Statistical Computing, Vienna, Austria) with madad and diagmeta packages.

#### Analysis of subgroups or subsets

We did not carry out any of the subgroup and additional outcome analyses planned in the protocol, due to the small number of studies or to the absence of the necessary information in study reports. For the same reasons, no sensitivity analysis was performed.

We assessed statistical heterogeneity for nonthreshold effect using *I*^2^ and the Cochrane *Q* test based on random effects analysis. *I*^2^ > 50% and the *p* value ≤ 0.05 were considered significant heterogeneity. For threshold effects, the heterogeneity was calculated by the visual inspection from the SROC curve [30–32].

## Results

### Study selection

**Fig. 1:**
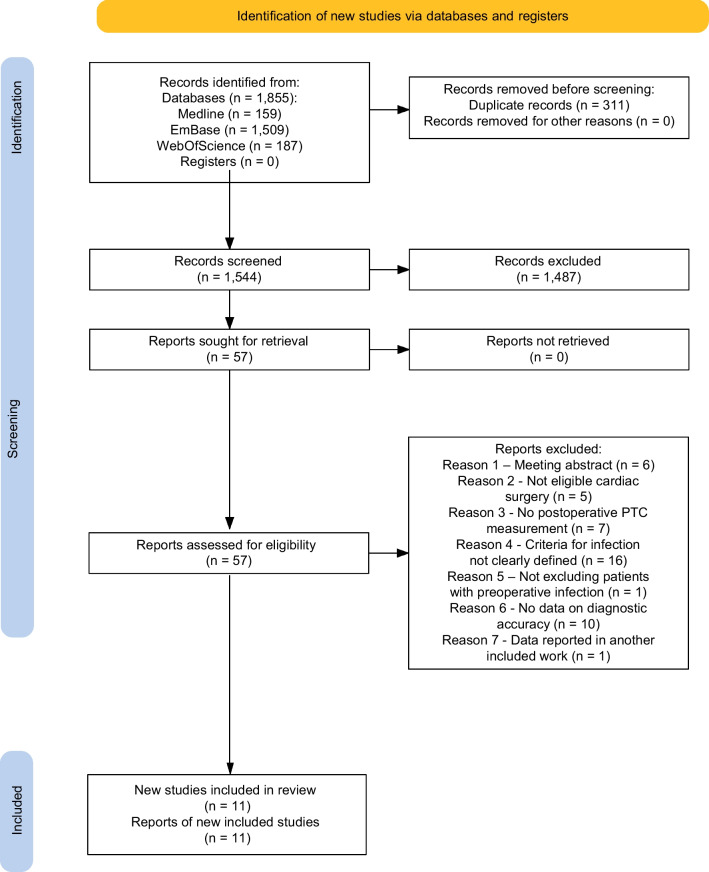
Study flow diagram

The PRISMA flow diagram for identification, screening, and inclusion of studies is shown in Fig. 1.

The original search performed on April 12th 2023 retrieved a total of 1855 records, which were uploaded into the Rayyan platform. After deduplication, 1544 records underwent manual title and abstract screening, of which 57 were identified as potentially eligible and underwent full text review. We excluded 46 reports [33–78] (see Additional file 2: Table S2), leaving 11 eligible studies which were included in our systematic review [17, 79–88]. Search rerun on September 15th, 2023 retrieved additional 130 deduplicated records, none of which was selected for full text review. Also, no additional eligible study was identified from reference lists of relevant papers.

### Study characteristics

Table 1 displays the characteristics of the 11 included studies. Overall 3803 patients (range from 40 to 819 per study) were involved.

**Table 1:** Characteristics of included studies in review

First author	Year	Study design	Patient sampling	Country	Timeframe	Single-center or multicenter	Main objective	Type of surgery	Timing of surgery	Clinical problem /Target condition	Reference standard (criteria for diagnosis of infection)	Independence of judgment for index test without knowing reference standard results	Independence of judgment for reference standard without knowing index test results	Index tests	Flow and timing of test	No. Patients	No. Patients with Infection vs no. Patients without infection
Chakravarthy [79]	2015	Observational with prospective data collection	Not specified	India	2013	Single-center	To determine the ability of elevated PCT levels to identify bacterial infections in cardiac surgical patients	Cardiac surgery	Elective	Infection	Positive cultures	Not specified	Not specified	PCT	Not specified	819	43 vs 776
Chen [80]	2022	Observational with prospective data collection	Consecutive	China	2020–2021	Single-center	To assess the value of IL-6 in the diagnosis of early pneumonia after CCH and compare it with that of PCT, CRP and WBC counts	Cardiac surgery with CPB	Elective	Pulmonary infection	Positive cultures, positive radiogram, clinical signs, laboratory findings	Not specified	Not specified	PCT, IL-6, CRP, and WBC count	Daily on POD 1 through 5	694	68 vs 626
de la Varga-Martínez [81]	2022	Observational with prospective data collection	Consecutive	Spain	2012–2016	Single-center	To evaluate the behavior of PCT and its usefulness in the diagnosis of postoperative pulmonary infection after Cardiac surgery in patients with or without impaired renal function	Heart valve surgery with CPB	Not specified	Pulmonary infection	Centers for Disease Control and Prevention definitions	Not specified	Not specified	PCT, CRP	ICU admission and at 8, 16, 24 and 72 h	803	42 vs 761
Jin [82]	2021	Observational with prospective data collection	Consecutive	China	2020–2021	Single-center	To investigate the ability of PCT variation to diagnose postoperative pneumonia after Cardiac surgery	Cardiac surgery with CPB	Elective	Pulmonary infection	Positive cultures, positive radiogram, clinical signs	Not specified	Two independent experts blinded to PCT, CRP and WBC count	PCT, WBC count and CRP	Before surgery, ICU admission, and daily on POD 1 through 5	272	24 vs 248
Li [83]	2021	Observational with retrospective data collection	Not specified	China	Not specified	Single-center	To investigate the early predictive value of PCT for the diagnosis of pulmonary infections after off-pump coronary artery bypass grafting	Off-pump coronary artery bypass grafting	Elective	Pulmonary infection	Centers for Disease Control and Prevention definitions	Not specified	Not specified	PCT, CRP, WBC	POD 1	131	23 vs 108
Liu [84]	2019	Observational with retrospective data collection	Consecutive	China	2015–2017	Single-center	To evaluate the value of PCT in diagnosing EPOP after off-pump CABG	First-time isolated off-pump CABG	Elective and urgent	Early postoperative pneumonia (within 3 days after CABG)	Positive cultures, positive radiogram, clinical signs, laboratory findings	Not specified	Not specified	PCT, WBC, neutral granulocyte ratio	POD 1	402	44 vs 358
Miao [17]	2022	Observational with retrospective data collection	Not specified	China	2019–2020	Single-center	To evaluate the value of dynamic monitoring of PCT as a biomarker for the early diagnosis of postoperative infections in patients undergoing cardiac surgery	Cardiac surgery with CPB	Not specified	Infection	Positive cultures	Not specified	Not specified	PCT	on POD 1, 3, and 5	210	41 vs 169
Sharma [85]	2016	Observational with prospective data collection	Consecutive	India	2015	Single-center	To compare the efficacy of PCT with WBC in predicting infection after CPB surgery. To assess the prognostic significance of PCT levels on the postoperative day 1	Cardiac surgery with CPB	Elective	Infection	Positive cultures	Not specified	Not specified	PCT, WBC count	POD 1	100	20 vs 80
Wang [86]	2017	Observational with retrospective data collection	Not specified	China	2014–2017	Single-center	To determine the value of PCT as an early marker of postoperative infection after Cardiac surgery with CPB	Cardiac surgery with CPB	Not specified	Infection	Centers for Disease Control and Prevention definitions	Not specified	Not specified	PCT	ICU admission, POD 1, 3, and 5	82	25 vs 57
Zhu [87]	2014	Observational with retrospective data collection	Not specified	China	2009–2012	Single-center	To investigate the changes in mean neutrophil volume before and after surgery, called ΔMNV; to compare the ΔMNV with PCT and CRP in terms of diagnostic sensitivity and specificity for postsurgical bacterial infection	Cardiac surgery	Not specified	Infection	Positive cultures, clinical signs, laboratory findings	Not specified	Not specified	PCT, CRP, Neutrophil CPD, WBC count and neutrophil percentage	POD 2 and 3	250	31 vs 219
Zou [88]	2018	Observational with prospective data collection	Consecutive	China	2017	Single-center	To explore the role of serum intestinal fatty acid-binding protein (IFABP) as a predictor of prognosis in postoperative Cardiac surgical patients	Heart valve surgery and/or coronary artery bypass graft	Not specified	Infection	Positive cultures, positive radiogram, clinical signs	Not specified	Not specified	PCT, IFABP	ICU admission	40	12 vs 28

*CRP* C-Reactive Protein, *WBC* White Blood Cell, *VAP* Ventilator-associated Pneumonia, *IL-6* interleukin-6, *IFABP* Intestinal Fatty Acid-Binding Protein, *ICU* Intensive Care Unit, *SIRS* Systemic Inflammatory Response Syndrome, *POD* PostOperative Day, *EPOP* Early Postoperative Pneumonia, *CABG* Coronary Artery Bypass Grafting surgery

All studies were single-center with observational design, five of which with retrospective data collection [17, 83, 84, 86, 87]. The vast majority was conducted in Asia (eight in China [17, 80, 82–84, 86–88], two in India [79, 85]), and only one in Europe [81].

The target condition was generically indicated as bacterial infection in six studies [17, 79, 85–88], whereas five studies focused exclusively on pulmonary infection [80–84]. The reference standards used to define infection varied. Three studies applied Centers for Disease Control (CDC) criteria [81, 83, 86], and the others all used positive cultures, either alone [17, 79, 85], or in combination with different parameters including cultures, imaging, laboratory findings, and clinical signs [80, 82, 84, 87, 88] (Table 1). Only one study did not report the technique adopted for measuring plasmatic PCT [88], while all other studies used the chemiluminescence immunoassay. However, only five studies provided information on the specific assay and its sensitivity range [79–81, 84, 87].

Timing of PCT measurement also varied, with four studies performing only one measurement, three studies on the first PostOperative Day (POD) [83–85], and the other at ICU admission [88]. The longest reported monitoring period was POD 5 in four studies [17, 80, 82, 86].

### Risk of bias assessment

**Fig. 2:**
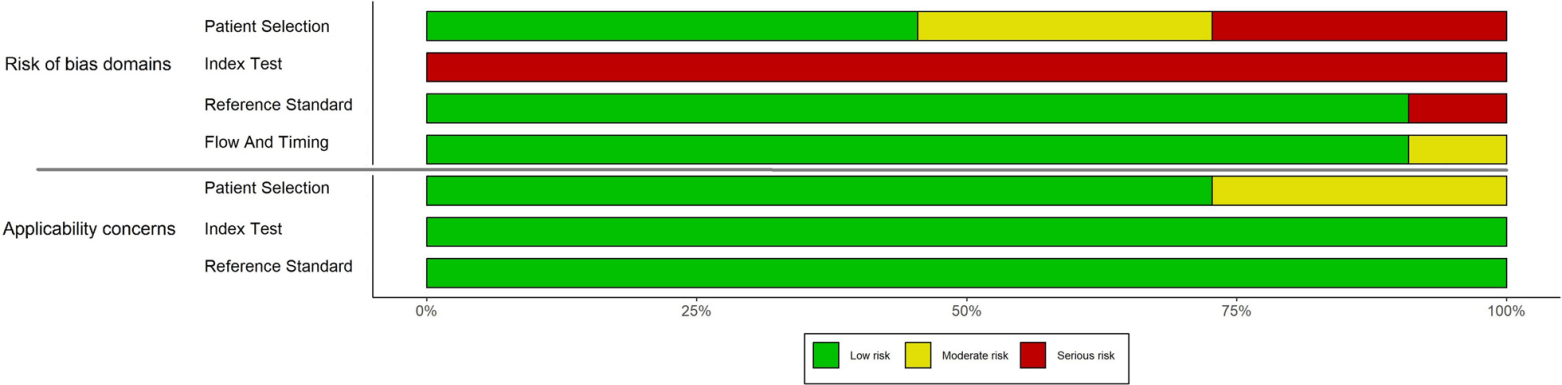
Risk of bias and applicability concerns graph: review authors' judgments about each domain presented as percentages across included studies

**Fig. 3:**
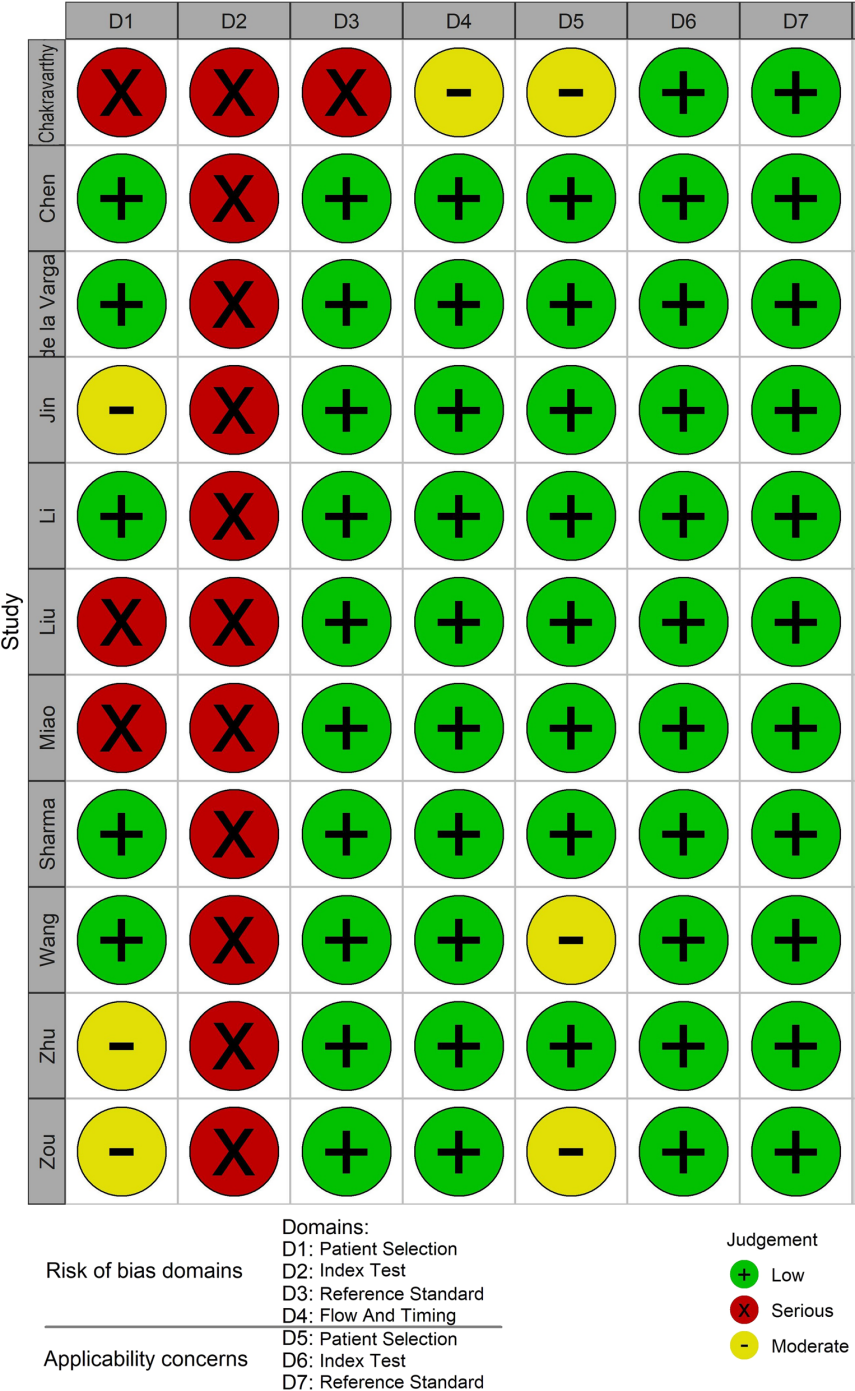
Risk of bias and applicability concerns summary: review authors' judgments about each domain for each included study

The methodological quality assessments with the QUADAS-2 tool results are summarized in Fig. 2 and further illustrated for individual studies in Fig. 3.

No study had a low risk of bias in all 4 domains. For the domain of risk of bias in patient selection, only five studies provided clear definitions of exclusion criteria and were judged as ‘low’ risk. Regarding the risk of bias for index tests, none of the studies prespecified a threshold and therefore they were all rated as ‘high risk’. Only one of the studies was judged to be at high risk of bias for the reference standard domain and for the patient flow and timing domain [79]. Seven studies were rated as ‘low’. Only three studies [79, 86, 88] were considered to have concerns about applicability, all in terms of patient selection. Further details on how judgments were made for each individual study are provided in Additional file 3: Table S3.

In the light of the issues that emerged from the risk of bias assessment, ten of the eleven studies were included in the meta-analysis. The study by Chakravarthy et al. [79] was excluded, because it exhibited high risk of bias in three domains and because it did not specify the execution time of the index test, making it impossible to attribute the outcome to a specific postoperative day.

### Overall accuracy of PCT

**Fig. 4:**
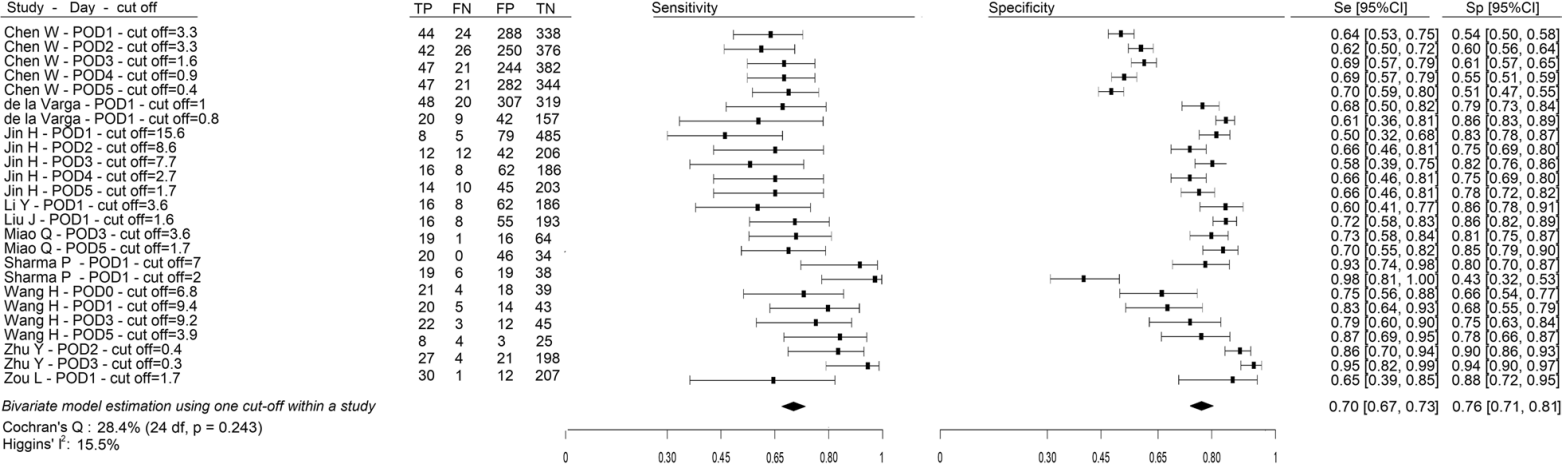
Forest plot of PCT diagnostic accuracy

Figure 4 shows the diagnostic accuracy of PCT in detecting bacterial infection after cardiac surgery, as reported in each of the 10 studies (2984 patients) included in the meta-analysis. The forest plots highlight the heterogeneity in test timing and in thresholds reported by each study, and in the corresponding values of Se and Sp and their 95%CI. The two diamonds represent, respectively, the pooled estimation of Se (0.70, 95%CI 0.67–0.73) and Sp (0.76, 95%CI 0.71–0.81). Concerning heterogeneity, through univariate analysis independent by thresholds, we determined values of *I*^2^ = 15.5 and *Q* = 28.4, which do not highlight significant heterogeneity (*p* = 0.243).

Concerning other diagnostic accuracy values, pooled median PLR, NLR and DOR of PCT were 2.96 (95%CI 2.33–3.74), 0.40 (95%CI 0.35–0.46), and 7.53 (95%CI 5.18–10.60), respectively. Based on the meaning attributed to the PLR value, a diseased patient is nearly three times more likely to have a positive test compared to a non-diseased patient; conversely, considering NLR, a non-diseased patient is 2.5 times more likely to have a negative test compared to a diseased patient. Furthermore, the value of DOR indicated that for PCT the odds for positivity among subjects with bacterial infection were nearly eight times higher than the odds for positivity among subjects without bacterial infections.

**Fig. 5:**
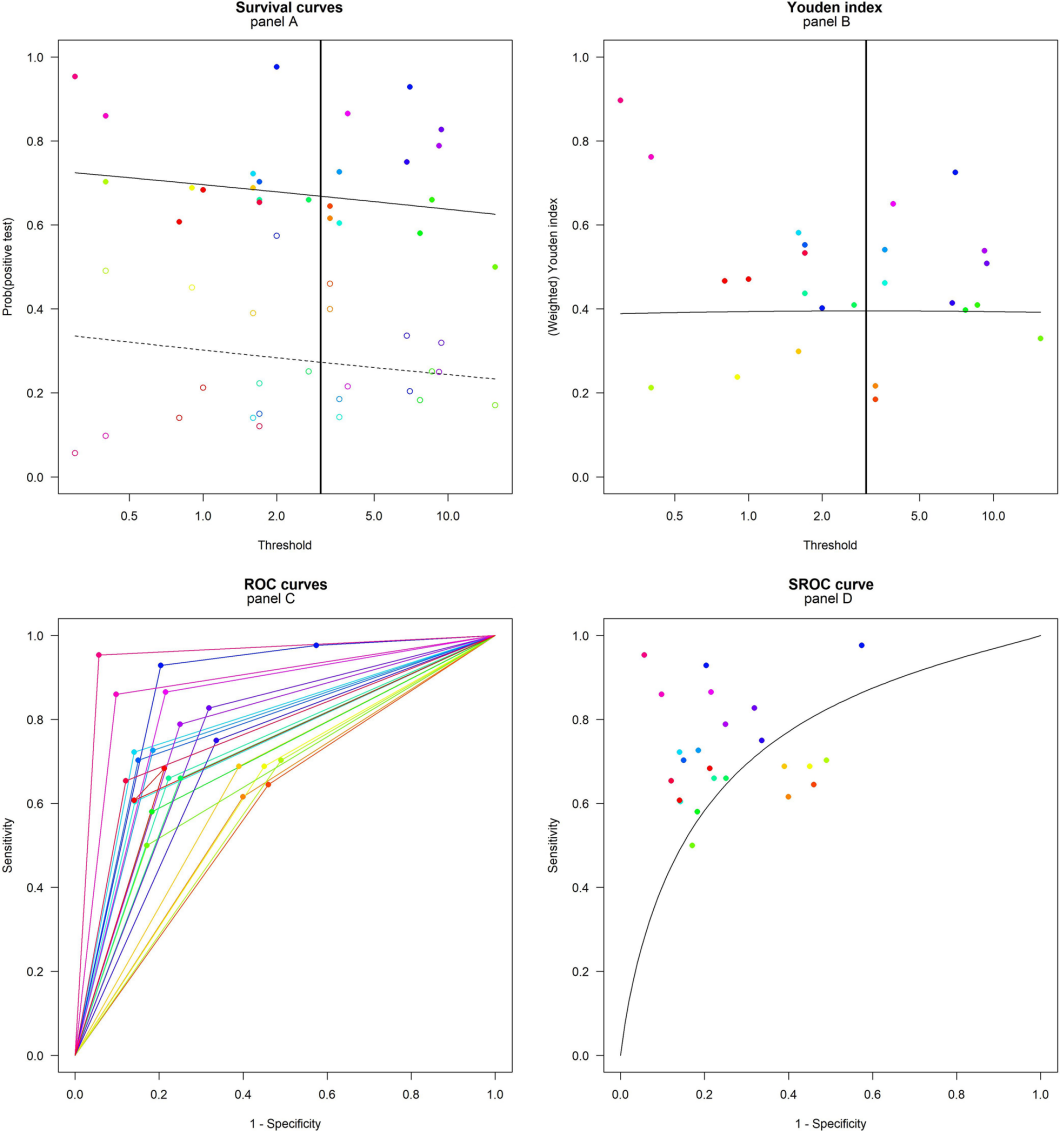
Summary receiver operating characteristic (SROC) curve (bivariate analysis using multiple thresholds within a study) for diagnostic test accuracy. Each color identifies a different study for individual POD

Results of bivariate model analysis using multiple thresholds within a study are depicted in Fig. 5.

The first two scatterplots from the top (panel A and B) show the optimal threshold as 3 ng/mL (with corresponding Se 0.67 (95%CI 0.47–0.82) and Sp 0.73 (95%CI 0.65–0.79)), which allows to best identify the diseased and non-diseased groups (solid and dashed lines) in terms of probability positive test and in terms of the corresponding maximum value of the Youden index.

The two lower scatterplots (panel C and D) display the individual ROC curves for each study and the SROC curve corresponding to the optimal threshold. The AUC of the SROC is of 0.75 (IC95% 0.29–0.95), which is considered to be “good” diagnostic accuracy [89] even though wide variability was observed.

**Fig. 6:**
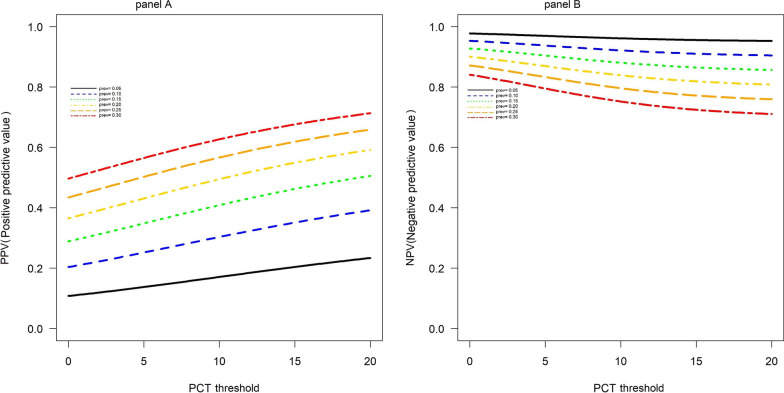
Plots illustrating corresponding **A** positive predictive values and **B** negative predictive values for different PCT threshold and prevalences, based on the multiple thresholds model

Table 2 reports performance measures, calculated considering prespecified ranges of thresholds and prevalence. Predictive values are further illustrated by continuous lines in Fig. 6, in which the threshold range is amplified (up to 20). As evident in Panel A, PPV varies approximately between 0.50 and 0.70, when prevalence is high (30%). Regarding NPV, the value is always > 90% when prevalence is < 20% (regardless of the threshold), and is reduced to 83% when prevalence is high (30%).

**Table 2:** Sensitivities and specificities at predefined thresholds and corresponding PPVs and NPVs for different prevalences, based on the multiple thresholds model

Threshold	Sensitivity	95%CI	Specificity	95%CI	Prevalence	PPV	NPV	FP*	FN*
1	0.7	0.47–0.85	0.7	0.58–0.8	0.05	0.11	0.98	29	1
					0.1	0.2	0.95	27	3
					0.15	0.29	0.93	26	4
					0.2	0.37	0.9	24	6
					0.25	0.43	0.87	23	7
					0.3	0.5	0.84	21	9
2	0.68	0.48–0.83	0.72	0.63–0.79	0.05	0.11	0.98	27	2
					0.1	0.21	0.95	25	3
					0.15	0.3	0.93	24	5
					0.2	0.37	0.9	22	6
					0.25	0.44	0.87	21	8
					0.3	0.51	0.84	20	10
3	0.67	0.47–0.82	0.73	0.65–0.79	0.05	0.11	0.98	26	2
					0.1	0.21	0.95	24	3
					0.15	0.3	0.93	23	5
					0.2	0.38	0.9	22	7
					0.25	0.45	0.87	20	8
					0.3	0.51	0.84	19	10
4	0.66	0.46–0.82	0.73	0.66–0.8	0.05	0.12	0.98	26	2
					0.1	0.22	0.95	24	3
					0.15	0.31	0.93	23	5
					0.2	0.38	0.9	22	7
					0.25	0.45	0.87	20	8
					0.3	0.52	0.84	19	10
5	0.66	0.45–0.82	0.74	0.67–0.8	0.05	0.12	0.98	25	2
					0.1	0.22	0.95	23	3
					0.15	0.31	0.92	22	5
					0.2	0.39	0.9	21	7
					0.25	0.46	0.87	20	8
					0.3	0.52	0.83	18	10

*FN* false negative, *FP* false positive, *NPV* negative predictive value, *PPV* positive predictive value

*Number of false positives and negatives in 100 hypothetical cases

**Fig. 7:**
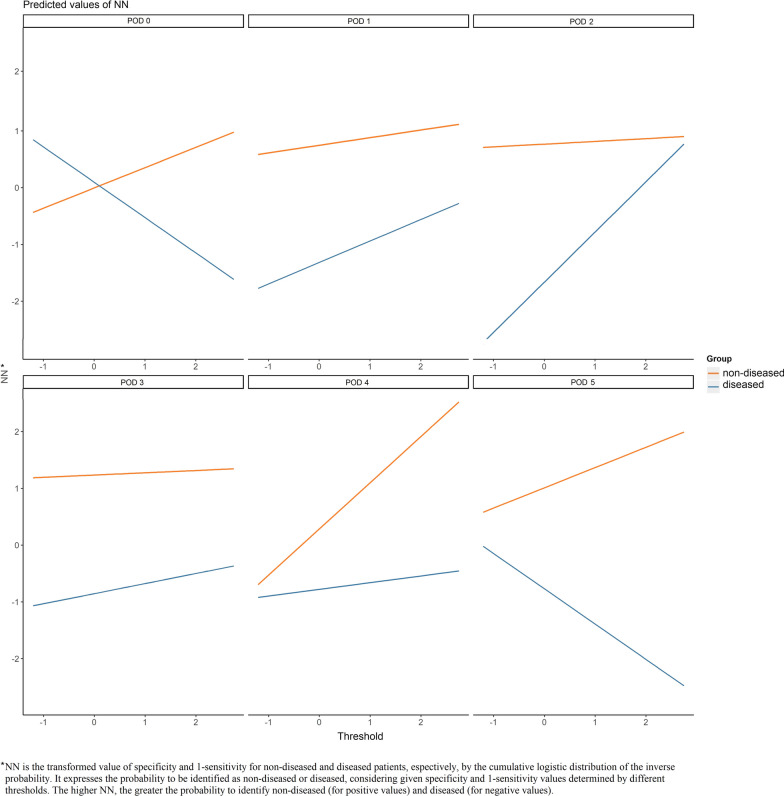
Interaction plot for different thresholds and for each POD. The lines represent diseased and non-diseased groups. The X axis reports unit increment/decrement of the threshold coefficient. variations

The results of the analysis where the interaction term threshold*day was included are displayed in Additional file 4: Table S4. The corresponding coefficient value is equal to − 0.24 (95%CI − 0.48 to 0.00), implying that the threshold should be decreased by 0.24 points per day. Although this finding is close to statistical significance (*p* = 0.053), for explorative purposes we examined it for each of the 6 PODs (Fig. 7). Starting from POD 1 to POD 4, the FN rate is reduced as the threshold decreases. This is especially true on POD 2, for which the finding is statistically significant (*p* = 0.019) (see Additional file 5: Table S5), identifying it as the probable best time point to use PCT for the diagnosis of infection.

## Discussion

Infection after cardiac surgery is a common complication but its timely diagnosis is challenging, since surgery, especially with the use of CPB, is a well-known trigger of systemic inflammation, producing biochemical and clinical patterns very similar to the ones observed during infection[5]. As a consequence, many markers of infection were shown to be unreliable in this condition [90].

### Main findings

To our knowledge, this is the first systematic review and meta-analysis investigating the role of PCT for the diagnosis of postoperative infection only including adult patients after cardiac surgery. Our meta-analysis, including 10 studies and 2984 patients, assessed the diagnostic test accuracy of PCT, considering different thresholds and different time points reported in included studies. Bivariate analysis using multiple thresholds within a study enabled us to highlight important characteristics of the diagnostic test. Specifically, we identified the optimal threshold value at 3 ng/mL, which is considerably higher than the 0.5 to 1.0 ng/mL range generally recommended for the diagnosis of postoperative infection[8]. However, even when considering this optimal threshold, test performance was limited, with a sensitivity of 67% and specificity of 73%. These findings may be due to the presence of systemic inflammation immediately after surgery, a hypothesis also supported by our analysis of the interaction between threshold and POD, which suggested that the threshold should be reduced daily to improve PCT diagnostic accuracy, and especially to increase the positive predictive value. Our analysis also suggested that POD 2 may be the best timing to diagnose infection with PCT, an indication also reported by other studies [82, 91]. Another interesting aspect worth noting, particularly relevant for clinical practice, is the test’s considerable ability to identify non-diseased individuals (NPV between 83 and 98%, with a prevalence range between 30 and 5%), and its poor utility in identifying diseased patients (PPV never exceeding 60%, even considering a high prevalence of 30%). This suggests that the use of procalcitonin in this context is useful to exclude, and not to confirm, the presence of a bacterial infection.

Concerning risk of bias assessment, various problems were detected. One of the main issue concerned the fact that threshold determination occurred a posteriori by ROC curve analysis in all studies, which may have led to optimistic test performance. Moreover, none of the studies was multicenter and none formally defined sample size a priori considering study endpoints.

Comparison of our results with other meta-analyses was not possible, because the only one published recently on this topic [16] considered both adults and children, and the analysis model used did not take into account the different thresholds reported in individual studies.

### Strengths and limitations

This systematic review was conducted following rigorous methodology, for search strategy development, evidence analysis and quality appraisal, involving a multiprofessional research team. One of the main strengths of this work lies in the advanced meta-analysis methods used to summarize data according to multiple threshold values in each study. Furthermore, the use of strict eligibility criteria for our review (clear definition of target condition diagnosis, only adult populations and only publications from the last 10 years) helped reduce heterogeneity, thus improving generalizability of results. In particular, the decision to apply a date restriction was due to the fact that perioperative standards of care (e.g. surgical techniques, extracorporeal circulation, Intensive Care Unit (ICU) care, etc.) have improved considerably in the last decade, leading to a reduction of surgery-related stress, and thus of SIRS, which may be misclassified as infection [92–94]. Although minimally invasive cardiac surgery, miniaturized and biocompatible CPB circuits, and fast-track protocols were all introduced over 20 years ago, their implementation has accelerated over the past decade [94–97].We also decided to exclude patients with transcatheter interventions, as these procedures are associated with a significantly lower degree of systemic inflammation, are usually performed on older, sicker patients, and could therefore impact on the generalizability of the results to the cardiac surgical population [98–100].

Some limitations of this work should also be acknowledged. Firstly, we only included studies that clearly indicated the diagnostic criteria applied to confirm infection, which may have lead to exclude relevant studies that did not report this aspect accurately. Unfortunately, we could not verify this potential bias with funnel plots, since this is not feasible in meta-analyses of diagnostic studies with multiple thresholds. Furthermore, the decision to apply a date restriction might have led to the exclusion of relevant studies. Secondly, included studies used different reference standards, which may have affected reliability of results. Furthermore, we acknowledge that although the analyzed literature aimed to exclude patients with preoperative infection, cases of undiagnosed preoperative infection cannot be ruled out, and this may have influenced results. Thirdly, in all studies, even when PCT measurements were taken on different days, the number of patients at risk considered for measuring test accuracy remained constant. This may have influenced the determination of the optimal threshold. Moreover, this prevented an unbiased estimation of the threshold for each POD. Finally, all included studies are observational, five of which with retrospective data collection, including one case–control study. This may have influenced reliability of results.

## Conclusions

This meta-analysis shows that in this target population, PCT performance is moderate, and accuracy good but not strong. Furthermore, the high NPV and low PPV values suggest the need for a paradigm shift in the use of PCT as a diagnostic marker for infection after cardiac surgery. In fact, while PCT is usually measured to confirm a suspected infection or as a screening tool in high risk populations, our results specific to individuals who underwent cardiac surgery suggest that for these patients it could rather be used to help exclude an infection that is deemed improbable. Another practical finding of this work is that a post-cardiac surgical PCT cutoff higher than that routinely employed in other aspects of clinical practice should be used. However, the optimal threshold of 3 ng/mL and time point of POD2 obtained in this meta-analysis need to be further investigated in large, well-designed randomized trials, aiming to establish whether health outcomes of patients receiving the test are better than those of patients who do not, corresponding to Phase IV diagnostic studies in the classification of Sackett and Haynes [101]. Only if robust evidence emerge, will it be possible to provide indications for clinical practice.

### Supplementary Information

**Additional file 1:** Search strategy.

**Additional file 2:** Studies excluded after full text review and corresponding reasons.

**Additional file 3:** Review authors’ judgements about each risk of bias item for each included study.

**Additional file 4:** Results Linear Mixed-Effects Models with interaction terms of Threshold × Group × POD.

**Additional file 5:** Results Linear Mixed-Effects Models with interaction terms of Threshold × Group × POD (as factor).

## Data Availability

The datasets used and/or analyzed during the current study are available from the corresponding author upon reasonable request.

## Abbreviations

AUC: Area Under the Curve
CI: Confidence Interval
CPB: Cardiopulmonary Bypass
DOR: Diagnostic Odds Ratio
FN: False Negative
FP: False Positive
ICU: Intensive Care Unit
NLR: Negative Likelihood Ratio
PCT: Procalcitonin
PLR: Positive Likelihood Ratio
POD: Postoperative Day
Se: Sensitivity
SIRS: Systemic Inflammatory Response Syndrome
Sp: Specificity
SROC: Summary Receiver Operating Characteristic
TN: True Negative
TP: True Positive
