# Supplementary material for: Procalcitonin for the diagnosis of postoperative bacterial infection after adult cardiac surgery: a systematic review and meta-analysis
Source: Crit Care. 2024 Feb 7;28:44. doi: 10.1186/s13054-024-04824-3 (PMC10848477; doi:10.1186/s13054-024-04824-3)
Supplement: Supplementary file 1 — Additional file 1: Search strategy. [file 13054_2024_4824_MOESM1_ESM.docx]

TABLE S1: SEARCH STRATEGY

**Notes on development**

The search strategies were developed by an information specialist closely working with the clinical team, and following the indications of the Cochrane Handbook for Systematic Reviews of Diagnostic Test Accuracy [1].

The strategy was piloted on PubMed in March 2023. As recommended in the Cochrane Handbook, no filters to detect diagnostic accuracy studies were used. We first tested a 3-component strategy, comprising strings for index test (Procalcitonin), population (cardiac surgery patients), and target condition (infection). We tested accuracy by comparing results with the references of the meta-analysis by Lee et al [2]. All studies included by Lee et al were captured only when the string for infection was not used (because some studies did not mention infection in the title and abstract, for instance generally referring to postoperative complications). We therefore decided to omit the string for infection. Also, no terms for reference tests were introduced, as they were not specified in the protocol. The final strategy therefore consisted in two strings, for indext test and population.

The PubMed search was then translated for use in EmBase and WebOfScience.

**String for PubMed**

(("Procalcitonin"[Mesh] OR procalcitonin[Title/Abstract] OR procalcytonin[Title/Abstract] OR Procalcitonine[Title/Abstract]) AND (("cardiac surgical procedures"[MeSH Terms] OR "Thoracic surgery"[MeSH Terms] OR "Cardiac surgery"[Title/Abstract] OR "Heart surgery"[Title/Abstract] OR "cardiac surgical procedures"[Title/Abstract] OR "Cardiac surgical procedure"[Title/Abstract] OR "Heart surgical procedures"[Title/Abstract] OR "heart surgical procedure"[Title/Abstract] OR "Coronary Artery Bypass"[MeSH Terms] OR "CABG"[Title/Abstract] OR "Coronary Artery Bypass"[Title/Abstract] OR "coronary artery by-pass"[Title/Abstract]) OR ("heart"[Title/Abstract] AND "surgery"[Title/Abstract]) OR ("cardiac"[Title/Abstract] AND "surgery"[Title/Abstract]) OR ("coronary"[Title/Abstract] AND "artery"[Title/Abstract] AND "bypass"[Title/Abstract]) OR ("coronary"[Title/Abstract] AND "artery"[Title/Abstract] AND "by-pass"[Title/Abstract]) OR ("Valv*"[Title/Abstract]) OR ("Mitral"[Title/Abstract] AND "surgery"[Title/Abstract]) OR ("Tricuspid"[Title/Abstract] AND "surgery"[Title/Abstract])))

**String for WebOfScience**

Procalcitonin OR Procalcytonin OR Procalcitonine

AND

"Cardiac surgery" OR "Heart surgery" OR "cardiac surgical procedures" OR "Cardiac surgical procedure" OR "Heart surgical procedures" OR "heart surgical procedure" OR "CABG" OR "Coronary Artery Bypass" OR "coronary artery by-pass" OR “Valv*” OR “Mitral surgery” OR “Tricuspid surgery”

*All terms were search in the “topic” field*

**String for EmBase**

('procalcitonin'/exp OR procalcitonin:ti,ab OR procalcytonin:ti,ab OR procalcitonine:ti,ab) AND ('heart surgery'/exp OR 'cardiovascular surgery'/exp OR 'thorax surgery'/exp OR 'cardiac surgery':ti,ab OR 'heart surgery':ti,ab OR 'cardiac surgical procedures':ti,ab OR 'cardiac surgical procedure':ti,ab OR 'heart surgical procedures':ti,ab OR 'heart surgical procedure':ti,ab OR 'coronary artery bypass graft'/exp OR 'cabg':ti,ab OR 'coronary artery bypass':ti,ab OR 'coronary artery by-pass':ti,ab OR ('heart':ti,ab AND 'surgery':ti,ab) OR ('cardiac':ti,ab AND 'surgery':ti,ab) OR ('coronary':ti,ab AND 'artery':ti,ab AND 'bypass':ti,ab) OR ('coronary':ti,ab AND 'artery':ti,ab AND 'by-pass':ti,ab) OR 'valv*':ti,ab OR ('mitral':ti,ab AND 'surgery':ti,ab) OR ('tricuspid':ti,ab AND 'surgery':ti,ab))

REFERENCES

1. Spijker R, Dinnes J, Glanville J, Eisinga A. Chapter 6: Searching for and selecting studies. Draft version (4 October 2022) for inclusion in: Deeks JJ, Bossuyt PM, Leeflang MM, Takwoingi Y, editor(s). Cochrane Handbook for Systematic Reviews of Diagnostic Test Accuracy Version 2. London: Cochrane].

2. Li Q, Zheng S, Zhou PY, Xiao Z, Wang R, Li J. The diagnostic accuracy of procalcitonin in infectious patients after cardiac surgery: a systematic review and meta-analysis. J Cardiovasc Med (Hagerstown). 2021 Apr 1;22(4):305-312. doi: 10.2459/JCM.0000000000001017.
