# Supplementary material for: Procalcitonin for the diagnosis of postoperative bacterial infection after adult cardiac surgery: a systematic review and meta-analysis
Source: Crit Care. 2024 Feb 7;28:44. doi: 10.1186/s13054-024-04824-3 (PMC10848477; doi:10.1186/s13054-024-04824-3)
Supplement: Supplementary file 2 — Additional file 2: Studies excluded after full text review and corresponding reasons. [file 13054_2024_4824_MOESM2_ESM.docx]

TABLE S2: STUDIES EXCLUDED AFTER FULL TEXT REVIEW AND CORRESPONDING REASONS

| First author | Year | Main reason for exclusion |
| --- | --- | --- |
| Amouzeshi A [33] | 2021 | Criteria for infection not clearly defined |
| Bauer A [34] | 2018 | Criteria for infection not clearly defined |
| Baumbach H [35] | 2016 | No postoperative PCT measurement |
| Baysal A [36] | 2015 | Meeting abstract |
| Boeken U [37] | 2019 | Meeting abstract |
| Brocca A [38] | 2017 | Criteria for infection not clearly defined |
| Brodska H [39] | 2018 | Not eligible surgery |
| Cheng ZB [40] | 2020 | Criteria for infection not clearly defined |
| Clementi A [41] | 2017 | Criteria for infection not clearly defined |
| Clementi A [42] | 2019 | No data on diagnostic accuracy |
| Cui J [43] | 2020 | No postoperative PCT measurement |
| Diab M [44] | 2019 | Meeting abstract |
| Dreymueller D [45] | 2016 | No data on diagnostic accuracy |
| Franeková J [46] | 2017 | No data on diagnostic accuracy |
| Hanafy DA [47] | 2021 | Criteria for infection not clearly defined |
| Heredia-Rodríguez M [48] | 2016 | No data on diagnostic accuracy |
| Heredia-Rodríguez M [49] | 2017 | No data on diagnostic accuracy |
| Hrazdilová O [50] | 2021 | Not eligible surgery |
| Imperiali CE [51] | 2020 | Criteria for infection not clearly defined |
| Jiao J [52] | 2015 | Not excluding patients with preoperative infection |
| Kettner J [53] | 2017 | Not eligible surgery |
| Klingele M [54] | 2015 | Criteria for infection not clearly defined |
| Klingele M [55] | 2016 | Criteria for infection not clearly defined |
| Kupiec A [56] | 2020 | Criteria for infection not clearly defined |
| Lagrost L [57] | 2014 | No postoperative PCT measurement |
| Laudisio A [58] | 2021 | No data on diagnostic accuracy |
| Liu H [59] | 2017 | No data on diagnostic accuracy |
| Ma B [60] | 2020 | Criteria for infection not clearly defined |
| Mitaka C [61] | 2013 | Not eligible surgery |
| Mlejnsky F [62] | 2015 | Not eligible surgery |
| Mohamed HE [63] | 2016 | Meeting abstract |
| Mony U [64] | 2019 | Meeting abstract |
| Nadziakiewicz P [65] | 2020 | Criteria for infection not clearly defined |
| Nemeth E [66] | 2018 | Criteria for infection not clearly defined |
| Pan T [67] | 2023 | No postoperative PCT measurement |
| Partylova EA [68] | 2019 | Criteria for infection not clearly defined |
| Pavalascu A [69] | 2015 | Meeting abstract |
| Perrotti A [70] | 2017 | No postoperative PCT measurement |
| Perrotti A [71] | 2018 | No data on diagnostic accuracy |
| Popov D [72] | 2015 | Criteria for infection not clearly defined |
| Saito J [73] | 2017 | No data on diagnostic accuracy |
| Schmidt T [74] | 2018 | No postoperative PCT measurement |
| Schoe A [75] | 2015 | No postoperative PCT measurement |
| Song YY [76] | 2020 | No data on diagnostic accuracy |
| Xie M [77] | 2022 | Data reported in another included work |
| Zhao D [78] | 2014 | Criteria for infection not clearly defined |
