# Supplementary material for: Procalcitonin for the diagnosis of postoperative bacterial infection after adult cardiac surgery: a systematic review and meta-analysis
Source: Crit Care. 2024 Feb 7;28:44. doi: 10.1186/s13054-024-04824-3 (PMC10848477; doi:10.1186/s13054-024-04824-3)
Supplement: Supplementary file 3 — Additional file 3: Review authors’ judgements about each risk of bias item for each included study. [file 13054_2024_4824_MOESM3_ESM.docx]

**TABLE S3: RISK OF BIAS SUMMARY: REVIEW AUTHORS’ JUDGEMENTS ABOUT EACH RISK OF BIAS ITEM FOR EACH INCLUDED STUDY**

| **Author** | **Domain 1: Participant selection**  **Yes/No/Unclear** | | | | | **Domain 2: Index test**  **Yes/No/Unclear** | | | | **Domain 3: Target condition and reference standard**  **Yes/No/Unclear** | | | | **Domain 4: Flow and timing**  **Yes/No/Unclear** | | | | |
| --- | --- | --- | --- | --- | --- | --- | --- | --- | --- | --- | --- | --- | --- | --- | --- | --- | --- | --- |
|  | Was a consecutive or random sample of patients enrolled? | Was a case-control design avoided? | Did the study avoid inappropriate exclusions? | Could the selection of patients have introduced bias?  **RISK:**  **Low/High**  **/Unclear** | Are there concerns that the included participants and setting do not match the review question?  **CONCERNS:**  **Low/High**  **/Unclear** | Were the index test results interpreted without knowledge of the results of the reference standard? | If a threshold was used, was it prespecified? | Could the conduct or interpretation of the index test have introduced bias?  **RISK:**  **Low/High**  **/Unclear** | Are there concerns that the index test, its conduct, or interpretation differ from the review question?  **CONCERNS:**  **Low/High**  **/Unclear** | Is the reference standard likely to correctly classify the target condition? | Were the reference standard results interpreted without knowledge of the results of the index test? | Could the reference standard, its conduct, or its interpretation have introduced bias?  **RISK:**  **Low/High**  **/Unclear** | Are there concerns that the target condition as defined by the reference standard does not match the question?  **CONCERNS:**  **Low/High**  **/Unclear** | Was there an appropriate interval between index test and reference standard? | Did all participants receive a reference standard? | Did all participants receive the same reference standard? | Were all participants included in the analysis? | Could the patient flow have introduced bias?  **RISK:**  **Low/High**  **/Unclear** |
| de la Varga Martínez O [81] | **Yes** | **Yes** | **Yes** | **Low** | **Low** | **Unclear** | **No**  Determined a posteriori by analysis of the ROC curve | **High**  Having optimized the threshold may have resulted in optimistic test performance | **Low** | **Yes** | **Unclear** | **Low** | **Low** | **Yes** | **Yes** | **Yes** | **Yes** | **Low** |

| **Author** | **Domain 1: Participant selection**  **Yes/No/Unclear** | | | | | **Domain 2: Index test**  **Yes/No/Unclear** | | | | **Domain 3: Target condition and reference standard**  **Yes/No/Unclear** | | | | **Domain 4: Flow and timing**  **Yes/No/Unclear** | | | | |
| --- | --- | --- | --- | --- | --- | --- | --- | --- | --- | --- | --- | --- | --- | --- | --- | --- | --- | --- |
|  | Was a consecutive or random sample of patients enrolled? | Was a case-control design avoided? | Did the study avoid inappropriate exclusions? | Could the selection of patients have introduced bias?  **RISK:**  **Low/High**  **/Unclear** | Are there concerns that the included participants and setting do not match the review question?  **CONCERNS:**  **Low/High**  **/Unclear** | Were the index test results interpreted without knowledge of the results of the reference standard? | If a threshold was used, was it prespecified? | Could the conduct or interpretation of the index test have introduced bias?  **RISK:**  **Low/High**  **/Unclear** | Are there concerns that the index test, its conduct, or interpretation differ from the review question?  **CONCERNS:**  **Low/High**  **/Unclear** | Is the reference standard likely to correctly classify the target condition? | Were the reference standard results interpreted without knowledge of the results of the index test? | Could the reference standard, its conduct, or its interpretation have introduced bias?  **RISK:**  **Low/High**  **/Unclear** | Are there concerns that the target condition as defined by the reference standard does not match the question?  **CONCERNS:**  **Low/High**  **/Unclear** | Was there an appropriate interval between index test and reference standard? | Did all participants receive a reference standard? | Did all participants receive the same reference standard? | Were all participants included in the analysis? | Could the patient flow have introduced bias?  **RISK:**  **Low/High**  **/Unclear** |
| Jin H [82] | **Yes** | **No**  Controls were age- and sex-matched | **Unclear**  “patients with missing clinical data (because of perioperative death or other reasons)” they don't say how many were excluded and there is no flow diagram. | **Unclear** | **Low** | **Unclear** | **No**  PCT were chosen to correspond to the best respective Youden’s index | **High**  Having optimized the threshold may have resulted in optimistic test performance | **Low** | **Yes**  The final diagnosis of PP was determined by 2 independent experts. | **Yes**  Experts were blinded to PCT | **Low** | **Low** | **Yes** | **Yes** | **Yes** | **Yes** | **Low** |

| **Author** | **Domain 1: Participant selection**  **Yes/No/Unclear** | | | | | **Domain 2: Index test**  **Yes/No/Unclear** | | | | **Domain 3: Target condition and reference standard**  **Yes/No/Unclear** | | | | **Domain 4: Flow and timing**  **Yes/No/Unclear** | | | | |
| --- | --- | --- | --- | --- | --- | --- | --- | --- | --- | --- | --- | --- | --- | --- | --- | --- | --- | --- |
|  | Was a consecutive or random sample of patients enrolled? | Was a case-control design avoided? | Did the study avoid inappropriate exclusions? | Could the selection of patients have introduced bias?  **RISK:**  **Low/High**  **/Unclear** | Are there concerns that the included participants and setting do not match the review question?  **CONCERNS:**  **Low/High**  **/Unclear** | Were the index test results interpreted without knowledge of the results of the reference standard? | If a threshold was used, was it prespecified? | Could the conduct or interpretation of the index test have introduced bias?  **RISK:**  **Low/High**  **/Unclear** | Are there concerns that the index test, its conduct, or interpretation differ from the review question?  **CONCERNS:**  **Low/High**  **/Unclear** | Is the reference standard likely to correctly classify the target condition? | Were the reference standard results interpreted without knowledge of the results of the index test? | Could the reference standard, its conduct, or its interpretation have introduced bias?  **RISK:**  **Low/High**  **/Unclear** | Are there concerns that the target condition as defined by the reference standard does not match the question?  **CONCERNS:**  **Low/High**  **/Unclear** | Was there an appropriate interval between index test and reference standard? | Did all participants receive a reference standard? | Did all participants receive the same reference standard? | Were all participants included in the analysis? | Could the patient flow have introduced bias?  **RISK:**  **Low/High**  **/Unclear** |
| Sharma P [85] | **Yes** | **Yes** | **Yes** | **Low** | **Low** | **Unclear** | **No**  Determined a posteriori by analysis of the ROC curve | **High**  Not having defined the threshold in advance, this could have led to optimistic test performances | **Low** | **Yes** | **Unclear** | **Low** | **Low** | **Yes** | **Yes** | **Yes** | **Yes** | **Low** |

| **Author** | **Domain 1: Participant selection**  **Yes/No/Unclear** | | | | | **Domain 2: Index test**  **Yes/No/Unclear** | | | | **Domain 3: Target condition and reference standard**  **Yes/No/Unclear** | | | | **Domain 4: Flow and timing**  **Yes/No/Unclear** | | | | |
| --- | --- | --- | --- | --- | --- | --- | --- | --- | --- | --- | --- | --- | --- | --- | --- | --- | --- | --- |
|  | Was a consecutive or random sample of patients enrolled? | Was a case-control design avoided? | Did the study avoid inappropriate exclusions? | Could the selection of patients have introduced bias?  **RISK:**  **Low/High**  **/Unclear** | Are there concerns that the included participants and setting do not match the review question?  **CONCERNS:**  **Low/High**  **/Unclear** | Were the index test results interpreted without knowledge of the results of the reference standard? | If a threshold was used, was it prespecified? | Could the conduct or interpretation of the index test have introduced bias?  **RISK:**  **Low/High**  **/Unclear** | Are there concerns that the index test, its conduct, or interpretation differ from the review question?  **CONCERNS:**  **Low/High**  **/Unclear** | Is the reference standard likely to correctly classify the target condition? | Were the reference standard results interpreted without knowledge of the results of the index test? | Could the reference standard, its conduct, or its interpretation have introduced bias?  **RISK:**  **Low/High**  **/Unclear** | Are there concerns that the target condition as defined by the reference standard does not match the question?  **CONCERNS:**  **Low/High**  **/Unclear** | Was there an appropriate interval between index test and reference standard? | Did all participants receive a reference standard? | Did all participants receive the same reference standard? | Were all participants included in the analysis? | Could the patient flow have introduced bias?  **RISK:**  **Low/High**  **/Unclear** |
| Chakravarthy M [79] | **Unclear** | **Yes** | **No**  “Patients were selected based on the presence of one or more of the criteria for diagnosing sepsis as described by Lever et al.[8]”  Furthermore, many exclusion criteria were applied, some of which do not appear to be justified, e.g. low ejection fraction and repeat or prolonged surgery. | **High**  For population eligibility the paper refers to a narrative synthesis of the definition and epidemiology of sepsis published in 2007, without specifying the criteria used. | **Unclear**  By only including patients with suspected sepsis, results may not be generalized to the target population of this review. | **Unclear** | **No** | **High**  It is not explained why they use various degrees of SPC level and where they come from (table 2). It all seems very arbitrary.  “Among the eleven patients in whom SPC levels were  suggestive of moderate sepsis and four patients in whom  the SPC value was suggestive of severe sepsis only one  had positive specimen culture” | **Low** | **No**  “The high degree of false  negativity might due to the method used in assessing  SPC levels; we use semi‑quantitative test to measure  SPC levels which gives false negative results” | **Unclear** | **High** | **Low** | **Unclear**  The timing of index test execution is not reported | **Yes** | **Yes** | **Yes** | **Unclear** |

| **Author** | **Domain 1: Participant selection**  **Yes/No/Unclear** | | | | | **Domain 2: Index test**  **Yes/No/Unclear** | | | | **Domain 3: Target condition and reference standard**  **Yes/No/Unclear** | | | | **Domain 4: Flow and timing**  **Yes/No/Unclear** | | | | |
| --- | --- | --- | --- | --- | --- | --- | --- | --- | --- | --- | --- | --- | --- | --- | --- | --- | --- | --- |
|  | Was a consecutive or random sample of patients enrolled? | Was a case-control design avoided? | Did the study avoid inappropriate exclusions? | Could the selection of patients have introduced bias?  **RISK:**  **Low/High**  **/Unclear** | Are there concerns that the included participants and setting do not match the review question?  **CONCERNS:**  **Low/High**  **/Unclear** | Were the index test results interpreted without knowledge of the results of the reference standard? | If a threshold was used, was it prespecified? | Could the conduct or interpretation of the index test have introduced bias?  **RISK:**  **Low/High**  **/Unclear** | Are there concerns that the index test, its conduct, or interpretation differ from the review question?  **CONCERNS:**  **Low/High**  **/Unclear** | Is the reference standard likely to correctly classify the target condition? | Were the reference standard results interpreted without knowledge of the results of the index test? | Could the reference standard, its conduct, or its interpretation have introduced bias?  **RISK:**  **Low/High**  **/Unclear** | Are there concerns that the target condition as defined by the reference standard does not match the question?  **CONCERNS:**  **Low/High**  **/Unclear** | Was there an appropriate interval between index test and reference standard? | Did all participants receive a reference standard? | Did all participants receive the same reference standard? | Were all participants included in the analysis? | Could the patient flow have introduced bias?  **RISK:**  **Low/High**  **/Unclear** |
| Zhu Y [87] | **Unclear** | **No**  Enrolling patients with known disease and a control group without the condition may exaggerate diagnostic accuracy | **Yes** | **Unclear** | **Low** | **Unclear** | **No**  Determined a posteriori by analysis of the ROC curve | **High**  Not having defined the threshold in advance, this could have led to optimistic test performances | **Low** | **Yes** | **Unclear** | **Low** | **Low** | **Yes** | **Yes** | **Yes** | **Yes** | **Low** |

| **Author** | **Domain 1: Participant selection**  **Yes/No/Unclear** | | | | | **Domain 2: Index test**  **Yes/No/Unclear** | | | | **Domain 3: Target condition and reference standard**  **Yes/No/Unclear** | | | | **Domain 4: Flow and timing**  **Yes/No/Unclear** | | | | |
| --- | --- | --- | --- | --- | --- | --- | --- | --- | --- | --- | --- | --- | --- | --- | --- | --- | --- | --- |
|  | Was a consecutive or random sample of patients enrolled? | Was a case-control design avoided? | Did the study avoid inappropriate exclusions? | Could the selection of patients have introduced bias?  **RISK:**  **Low/High**  **/Unclear** | Are there concerns that the included participants and setting do not match the review question?  **CONCERNS:**  **Low/High**  **/Unclear** | Were the index test results interpreted without knowledge of the results of the reference standard? | If a threshold was used, was it prespecified? | Could the conduct or interpretation of the index test have introduced bias?  **RISK:**  **Low/High**  **/Unclear** | Are there concerns that the index test, its conduct, or interpretation differ from the review question?  **CONCERNS:**  **Low/High**  **/Unclear** | Is the reference standard likely to correctly classify the target condition? | Were the reference standard results interpreted without knowledge of the results of the index test? | Could the reference standard, its conduct, or its interpretation have introduced bias?  **RISK:**  **Low/High**  **/Unclear** | Are there concerns that the target condition as defined by the reference standard does not match the question?  **CONCERNS:**  **Low/High**  **/Unclear** | Was there an appropriate interval between index test and reference standard? | Did all participants receive a reference standard? | Did all participants receive the same reference standard? | Were all participants included in the analysis? | Could the patient flow have introduced bias?  **RISK:**  **Low/High**  **/Unclear** |
| Chen W [80] | **Yes** | **Yes** | **Unclear**  It is not clear why pregnant or lactating women are excluded, however given the low fequency of this population the impact should be negligible | **Low** | **Low** | **Unclear** | **No**  Determined a posteriori by analysis of the ROC curve | **High**  Not having defined the threshold in advance, this could have led to optimistic test performances | **Low** | **Yes** | **Unclear** | **Low** | **Low** | **Yes** | **Yes** | **Yes** | **Yes** | **Low** |

| **Author** | **Domain 1: Participant selection**  **Yes/No/Unclear** | | | | | **Domain 2: Index test**  **Yes/No/Unclear** | | | | **Domain 3: Target condition and reference standard**  **Yes/No/Unclear** | | | | **Domain 4: Flow and timing**  **Yes/No/Unclear** | | | | |
| --- | --- | --- | --- | --- | --- | --- | --- | --- | --- | --- | --- | --- | --- | --- | --- | --- | --- | --- |
|  | Was a consecutive or random sample of patients enrolled? | Was a case-control design avoided? | Did the study avoid inappropriate exclusions? | Could the selection of patients have introduced bias?  **RISK:**  **Low/High**  **/Unclear** | Are there concerns that the included participants and setting do not match the review question?  **CONCERNS:**  **Low/High**  **/Unclear** | Were the index test results interpreted without knowledge of the results of the reference standard? | If a threshold was used, was it prespecified? | Could the conduct or interpretation of the index test have introduced bias?  **RISK:**  **Low/High**  **/Unclear** | Are there concerns that the index test, its conduct, or interpretation differ from the review question?  **CONCERNS:**  **Low/High**  **/Unclear** | Is the reference standard likely to correctly classify the target condition? | Were the reference standard results interpreted without knowledge of the results of the index test? | Could the reference standard, its conduct, or its interpretation have introduced bias?  **RISK:**  **Low/High**  **/Unclear** | Are there concerns that the target condition as defined by the reference standard does not match the question?  **CONCERNS:**  **Low/High**  **/Unclear** | Was there an appropriate interval between index test and reference standard? | Did all participants receive a reference standard? | Did all participants receive the same reference standard? | Were all participants included in the analysis? | Could the patient flow have introduced bias?  **RISK:**  **Low/High**  **/Unclear** |
| Zou L [88] | **Yes** | **Yes** | **Unclear**  It is not clear why patients > 75 years old were excluded. Moreover, patients with chronic digestive disease or previous gastrointestinal surgery are excluded; this criterion is justified by the study’s objective but may reduce result generalizability | **Unclear** | **Unclear**  The primary objective of the study is to explore the prognostic role of a different marker | **Unclear** | **No**  Determined a posteriori | **High**  Not having defined the threshold in advance, this could have led to optimistic test performances | **Low** | **Yes** | **Unclear** | **Low** | **Low** | **Yes** | **Yes** | **Yes** | **Yes** | **Low** |

| **Author** | **Domain 1: Participant selection**  **Yes/No/Unclear** | | | | | **Domain 2: Index test**  **Yes/No/Unclear** | | | | **Domain 3: Target condition and reference standard**  **Yes/No/Unclear** | | | | **Domain 4: Flow and timing**  **Yes/No/Unclear** | | | | |
| --- | --- | --- | --- | --- | --- | --- | --- | --- | --- | --- | --- | --- | --- | --- | --- | --- | --- | --- |
|  | Was a consecutive or random sample of patients enrolled? | Was a case-control design avoided? | Did the study avoid inappropriate exclusions? | Could the selection of patients have introduced bias?  **RISK:**  **Low/High**  **/Unclear** | Are there concerns that the included participants and setting do not match the review question?  **CONCERNS:**  **Low/High**  **/Unclear** | Were the index test results interpreted without knowledge of the results of the reference standard? | If a threshold was used, was it prespecified? | Could the conduct or interpretation of the index test have introduced bias?  **RISK:**  **Low/High**  **/Unclear** | Are there concerns that the index test, its conduct, or interpretation differ from the review question?  **CONCERNS:**  **Low/High**  **/Unclear** | Is the reference standard likely to correctly classify the target condition? | Were the reference standard results interpreted without knowledge of the results of the index test? | Could the reference standard, its conduct, or its interpretation have introduced bias?  **RISK:**  **Low/High**  **/Unclear** | Are there concerns that the target condition as defined by the reference standard does not match the question?  **CONCERNS:**  **Low/High**  **/Unclear** | Was there an appropriate interval between index test and reference standard? | Did all participants receive a reference standard? | Did all participants receive the same reference standard? | Were all participants included in the analysis? | Could the patient flow have introduced bias?  **RISK:**  **Low/High**  **/Unclear** |
| Wang H [86] | **Unclear** | **Yes** | **Yes** | **Low** | **Unclear**  By only including patients with SIRS, results may not b generalized to the target population of this review. | **Unclear** | **No**  Determined a posteriori | **High**  Not having defined the threshold in advance, this could have led to optimistic test performances | **Low** | **Yes** | **Unclear** | **Low** | **Low** | **Yes** | **Yes** | **Yes** | **Yes** | **Low** |

| **Author** | **Domain 1: Participant selection**  **Yes/No/Unclear** | | | | | **Domain 2: Index test**  **Yes/No/Unclear** | | | | **Domain 3: Target condition and reference standard**  **Yes/No/Unclear** | | | | **Domain 4: Flow and timing**  **Yes/No/Unclear** | | | | |
| --- | --- | --- | --- | --- | --- | --- | --- | --- | --- | --- | --- | --- | --- | --- | --- | --- | --- | --- |
|  | Was a consecutive or random sample of patients enrolled? | Was a case-control design avoided? | Did the study avoid inappropriate exclusions? | Could the selection of patients have introduced bias?  **RISK:**  **Low/High**  **/Unclear** | Are there concerns that the included participants and setting do not match the review question?  **CONCERNS:**  **Low/High**  **/Unclear** | Were the index test results interpreted without knowledge of the results of the reference standard? | If a threshold was used, was it prespecified? | Could the conduct or interpretation of the index test have introduced bias?  **RISK:**  **Low/High**  **/Unclear** | Are there concerns that the index test, its conduct, or interpretation differ from the review question?  **CONCERNS:**  **Low/High**  **/Unclear** | Is the reference standard likely to correctly classify the target condition? | Were the reference standard results interpreted without knowledge of the results of the index test? | Could the reference standard, its conduct, or its interpretation have introduced bias?  **RISK:**  **Low/High**  **/Unclear** | Are there concerns that the target condition as defined by the reference standard does not match the question?  **CONCERNS:**  **Low/High**  **/Unclear** | Was there an appropriate interval between index test and reference standard? | Did all participants receive a reference standard? | Did all participants receive the same reference standard? | Were all participants included in the analysis? | Could the patient flow have introduced bias?  **RISK:**  **Low/High**  **/Unclear** |
| Miao Q [17] | **Unclear** | **Yes** | **No**  The study excludes 245 (38.1%) patients “with incomplete clinical operation record information and no dynamic monitoring of PCT after surgery”. | **High** | **Low** | **Unclear** | **No**  Determined a posteriori | **High**  Not having defined the threshold in advance, this could have led to optimistic test performances | **Low** | **Yes** | **Unclear** | **Low** | **Low** | **Yes** | **Yes** | **Yes** | **Yes** | **Low** |

| **Author** | **Domain 1: Participant selection**  **Yes/No/Unclear** | | | | | **Domain 2: Index test**  **Yes/No/Unclear** | | | | **Domain 3: Target condition and reference standard**  **Yes/No/Unclear** | | | | **Domain 4: Flow and timing**  **Yes/No/Unclear** | | | | |
| --- | --- | --- | --- | --- | --- | --- | --- | --- | --- | --- | --- | --- | --- | --- | --- | --- | --- | --- |
|  | Was a consecutive or random sample of patients enrolled? | Was a case-control design avoided? | Did the study avoid inappropriate exclusions? | Could the selection of patients have introduced bias?  **RISK:**  **Low/High**  **/Unclear** | Are there concerns that the included participants and setting do not match the review question?  **CONCERNS:**  **Low/High**  **/Unclear** | Were the index test results interpreted without knowledge of the results of the reference standard? | If a threshold was used, was it prespecified? | Could the conduct or interpretation of the index test have introduced bias?  **RISK:**  **Low/High**  **/Unclear** | Are there concerns that the index test, its conduct, or interpretation differ from the review question?  **CONCERNS:**  **Low/High**  **/Unclear** | Is the reference standard likely to correctly classify the target condition? | Were the reference standard results interpreted without knowledge of the results of the index test? | Could the reference standard, its conduct, or its interpretation have introduced bias?  **RISK:**  **Low/High**  **/Unclear** | Are there concerns that the target condition as defined by the reference standard does not match the question?  **CONCERNS:**  **Low/High**  **/Unclear** | Was there an appropriate interval between index test and reference standard? | Did all participants receive a reference standard? | Did all participants receive the same reference standard? | Were all participants included in the analysis? | Could the patient flow have introduced bias?  **RISK:**  **Low/High**  **/Unclear** |
| Li Y [83] | **Unclear** | **Yes** | **Yes** | **Low** | **Low** | **Unclear** | **No**  Determined a posteriori | **High**  Not having defined the threshold in advance, this could have led to optimistic test performances | **Low** | **Yes** | **Unclear** | **Low** | **Low** | **Yes** | **Yes** | **Yes** | **Yes** | **Low** |

| **Author** | **Domain 1: Participant selection**  **Yes/No/Unclear** | | | | | **Domain 2: Index test**  **Yes/No/Unclear** | | | | **Domain 3: Target condition and reference standard**  **Yes/No/Unclear** | | | | **Domain 4: Flow and timing**  **Yes/No/Unclear** | | | | |
| --- | --- | --- | --- | --- | --- | --- | --- | --- | --- | --- | --- | --- | --- | --- | --- | --- | --- | --- |
|  | Was a consecutive or random sample of patients enrolled? | Was a case-control design avoided? | Did the study avoid inappropriate exclusions? | Could the selection of patients have introduced bias?  **RISK:**  **Low/High**  **/Unclear** | Are there concerns that the included participants and setting do not match the review question?  **CONCERNS:**  **Low/High**  **/Unclear** | Were the index test results interpreted without knowledge of the results of the reference standard? | If a threshold was used, was it prespecified? | Could the conduct or interpretation of the index test have introduced bias?  **RISK:**  **Low/High**  **/Unclear** | Are there concerns that the index test, its conduct, or interpretation differ from the review question?  **CONCERNS:**  **Low/High**  **/Unclear** | Is the reference standard likely to correctly classify the target condition? | Were the reference standard results interpreted without knowledge of the results of the index test? | Could the reference standard, its conduct, or its interpretation have introduced bias?  **RISK:**  **Low/High**  **/Unclear** | Are there concerns that the target condition as defined by the reference standard does not match the question?  **CONCERNS:**  **Low/High**  **/Unclear** | Was there an appropriate interval between index test and reference standard? | Did all participants receive a reference standard? | Did all participants receive the same reference standard? | Were all participants included in the analysis? | Could the patient flow have introduced bias?  **RISK:**  **Low/High**  **/Unclear** |
| Liu J [84] | **Yes** | **Yes** | **No**  The study excluded patients with “death within 3 days after surgery, or incomplete clinical data”. | **High** | **Low** | **Unclear** | **No**  Determined a posteriori | **High**  Not having defined the threshold in advance, this could have led to optimistic test performances | **Low** | **Yes** | **Unclear** | **Low** | **Low** | **Yes** | **Yes** | **Yes** | **Yes** | **Low** |
